# Supplementary material for: Development of an artificial intelligence-based algorithm to classify images acquired with an intraoral scanner of individual molar teeth into three categories
Source: PLoS One. 2022 Jan 7;17(1):e0261870. doi: 10.1371/journal.pone.0261870 (PMC8741029; doi:10.1371/journal.pone.0261870)
Supplement: Attachment — Submitted filename: Response to Reviewers.docx [file pone.0261870.s001.docx]

**Manuscript ID: PONE-D-21-30844**

Title: Development of an artificial intelligence-based algorithm to classify images acquired with an intraoral scanner of individual molar teeth into three categories

Point-by-Point Responses

We thank the reviewers for their careful reading of our manuscript and useful comments. Our responses to the reviewers’ comments are presented below. All revisions to the manuscript are highlighted in red-colored font and referred to by line number in the responses below. Please note that the changes made do not majorly affect the content, conclusions or framework of the paper.

**REVIEWER #1**

INTRODUCTION

*Comment*

The Introduction section should be improved better clarifying the potentiality and the novelty of the proposed approach, in order to classify it in relation to the most used techniques.

*Response*

Thank you for this excellent recommendation. We have completely rewritten the Introduction section to streamline the text and focus on the potentiality and novelty of our study in relation to currently used approaches. We refer you to the new Introduction section in the revised manuscript (lines 48–76).

MATERIALS AND METHODS

*Comment*

The ‘Intraoral 3D scanning’ section describes mainly the characteristics of the scanner, but there is no information on the experimental setup and procedure followed for the data acquisition from cadavers. In the Discussion section it is stated that the intraoral 3D scanning can be used also in challenging situations, typical when the mouth opening width is limited because of rigor mortis. For this reason, in the ‘Materials and Methods’ section, a step-by-step description of the procedure used for the 3D scanning acquisitions on cadavers should be reported, also highlighting, if there have been, difficulties faced.

*Response*

Thank you for this helpful suggestion. We have modified the Intraoral Scanning subsection of the Materials and Methods section to include more information about the technique used for intraoral scanning of cadavers (lines 96–105). We did not encounter any specific difficulties while performing intraoral scanning, hence none are described in the manuscript.

*Comment*

Line 89: dcm is the file extension. The file format is called DICOM.

*Response*

Thank you for pointing out this inadvertent error. We have revised “DCM” to “DICOM” (line 90).

*Comment*

Line 93: how many cadavers were available for the analysis?

*Response*

A total of 34 cadavers were available for our analysis. We have added this information to the Intraoral Scanning subsection of the Materials and Methods section (line 94).

*Comment*

Figure 1: this figure shows an example of the three molar teeth types, but for the model implementation it is stated that you have used scans. So, the reviewer is wondering if these scans were exported in some CAD format (usually stl) and then these used for the analysis. This point is not clear. For this reason, the reviewer suggests to show some images representing DICOM or stl models (or better both of them), in order to clarify the type of available data. Moreover, if some scans post-processing was performed this should be reported in the Methods section as well as the accuracy of the model (number of elements, texture, and so on).

*Response*

Thank you for these important queries. The images shown in Figure 1 were obtained as follows. First, the 3D images obtained from cadavers by intraoral scanning were saved as STL files, and occlusal views of the teeth were generated with a snipping tool and saved as PNG files. Then, ImageJ was used to generate individual images of the upper and lower molars (256×256 pixels) from the occlusal views of the teeth; examples of these images are presented in Figure 1. We have revised the Intraoral Scanning subsection of the Materials and Methods section to include the above information (lines 101–105). In addition, Figure 1 has been modified to include a panel showing an occlusal view generated by intraoral scanning, from which images of individual molar teeth were extracted. The title and legend for Figure 1 have been updated accordingly (lines 107–111).

*Comment*

Lines 126-127: here the authors stated that the number of available scanning from cadavers was not sufficient for the training of the network. Is it possible to sustain this with some bibliography and provide information on the proper number of training data?

*Response*

The number of images required to train a network varies depending on the structure of the network and the complexity of the problem. Currently, no methods are available to predict the number of images needed, and there are no relevant publications that we can cite. Since only a limited number of cadavers were available to us, we made the decision to use transfer learning to overcome any potential problems that might be associated with the use of a small training dataset. We have added some information about the transfer learning technique and a supporting reference citation to the Generation Of A Pre-trained Model subsection of the Materials and Methods section (lines 136–139).

*Comment*

Figure 4: this image is not very useful; indeed, the two-step cascade process is clear as it is explained in this section. It would be more beneficial for the reader if a comprehensive workflow of the overall process and AI-based algorithm is provided with all the main steps clearly described.

*Response*

Thank you for this helpful suggestion. We have modified Figure 4 to make it easier to understand.

*Comment*

Lines 167-168: this sentence in not comprehensible; please rephrase.

*Response*

We are sorry that the meaning of the original text was unclear. We have modified this sentence (lines 178–179).

**DISCUSSION**

*Comment*

Lines 201-202: It is not clear which features have been used for the identification of these conditions: texture, shape, volume? In the Methods section a more detailed description of the available features should be provided along with the features used within the AI architecture.

*Response*

We are sorry that the point was unclear. We assume that this was based on the color of the tooth in the image, because a metallic restoration appears darker in the image. However, we did not use a color threshold to distinguish dark metallic regions from light non-metallic regions because good accuracy was detected without setting. Therefore, there is no additional information at this stage.

*Comment*

Lines 205-206: How the model can be used for victim recognition based only on the recognition of the molar teeth condition? This point is not clear to the reviewer. The authors have described the full process for the creation and training of the network but its application for the specific case of victim recognition remains vague.

*Response*

Thank you for this important question. The long-term aim of this research is to develop an AI-based algorithm that can automatically generate a dental chart from images acquired with an intraoral scanner. These postmortem dental charts could be compared with antemortem records to facilitate the identification of disaster victims. As a first step toward this aim, the present study has developed an algorithm to classify the condition of the molar teeth into three types. We have completely rewritten the Introduction section (see lines 49–76) and Discussion section (lines 211–264) to better explain the objective, significance and future potential of our research.

*Comment*

Lines 233-234: the possible use of morphological characteristics for victim recognition is undoubtedly an improvement for the model, but how do you link the work here presented with the possibility to use new features for victim recognition? In general, the connection between the part of the work here presented and the overall project has to be better clarified and carried on throughout the paper.

*Response*

Since the focus of the present study was the detection of dental restorations (specifically, metallic restorations) rather than morphological characteristics, we have deleted the text referring to morphological characteristics in order to avoid confusion. Furthermore, we have rewritten the Discussion section to explain more clearly that the present research is a first step toward developing an automated system for the construction of postmortem dental charts from imaging data obtained by intraoral scanning.

**REVIEWER #2**

*Comment*

The abstract should be reworded and more clearly structured.

*Response*

Thank you for this helpful recommendation. We have rewritten the Abstract section of the manuscript to improve its structure (lines 20–46).

*Comment*

For the readers unfamiliar with intraoral scanners more information should be provided on the applied scanner and explanation why it is powder free should be added.

*Response*

Thank you for this useful suggestion. We have added more information about the intraoral scanner to the Intraoral Scanning subsection of the Materials and Methods section (lines 96–105) and the Discussion section (lines 222–234).

*Comment*

The main limitation of the study is a relatively low number of cases, especially from the intraoral scanner. However, preliminary results are promising.

*Response*

Since there was a limitation to the number of cadavers available for this research, we utilized the technique of transfer learning, which focuses on transferring the knowledge across domains to overcome the problems of small training datasets. Using this approach, we were able to develop our algorithm successfully despite the limited number of cadavers. We have added more details about the use of the transfer learning to the Generation Of A Pre-trained Model subsection of the Materials and Methods section (lines 1376–146) and to the Discussion section of the manuscript (lines 235­–243).

*Comment*

Another limitation of the study is that the authors teach the algorithm to distinguish between 3 groups: 1. full metallic crown 2. partial metallic restoration and 3. sound tooth, caries tooth and tooth with non-metallic restoration merged in one group. Probably metallic restorations are prevalent in the studied Japanese population, but this is a major limitation in populations in which more esthetic restorations are applied. The authors should discuss this limitation more in depth and also provide an attempt on solution of the problem of differentiation between healthy teeth and restored teeth using composite materials mimicking sound tissues.

*Response*

We agree entirely that the algorithm will need to be further developed to detect non-metallic (composite resin) restorations before it can be widely used to facilitate personal identification in the event of a large-scale disaster. The use of dental evidence for the personal identification of disaster victims requires that the collected dental findings are highly accurate. Since teeth with composite resin restorations can sometimes be mistaken for non-restored teeth even when observed with the naked eye, the present study limited the classification of molar tooth condition to only three categories (i.e., FMC, In and CNMR) that exhibit obvious differences in color. The accuracy of the algorithm was around 95% when these three categories were used and reached about 98% when the classification was based only on the presence or absence of a metal-colored restoration. We view our research as a first step toward the creation of a more complex algorithm that can accurately detect a wide range of tooth features, including both metallic and non-metallic restorations. Furthermore, we envisage that such an algorithm would allow postmortem dental charts to be automatically and rapidly generated from occlusal views obtained by intraoral scanning. We have completely rewritten the entire Discussion section of the manuscript, which now includes the above information (lines 243–257).

*Comment*

What did the authors mean by "treatment scars"?

*Response*

Thank you for this query. This term was intended to describe evidence of prior treatment. However, this term has been deleted from the manuscript following major revisions to the Discussion section.

*Comment*

The conclusions in the present form are rather an extended summary of the results, therefore should be rephrased so that they are related to the aim of the study.

*Response*

We have rewritten the conclusion so that it is better related to the aim of the study (lines 258–264).
