# Supplementary material for: Development of an artificial intelligence-based algorithm to classify images acquired with an intraoral scanner of individual molar teeth into three categories
Source: PLoS One. 2022 Jan 7;17(1):e0261870. doi: 10.1371/journal.pone.0261870 (PMC8741029; doi:10.1371/journal.pone.0261870)
Supplement: Attachment — Submitted filename: Response to Reviewers.docx [file pone.0261870.s002.docx]

**Manuscript ID: PONE-D-21-30844R1**

Title: Development of an artificial intelligence-based algorithm to classify images acquired with an intraoral scanner of individual molar teeth into three categories

Point-by-Point Responses

We thank the reviewer for his/her careful reading of our manuscript and useful comments. Our responses to the reviewers’ comments are presented below. All revisions to the manuscript are highlighted in red-colored font and referred to by line number in the responses below. Please note that the changes made do not majorly affect the content, conclusions or framework of the paper.

**REVIEWER #1**

*Comment*

The authors have addressed all the comments and now the paper is more detailed and sounds clearer for the reader.

*Response*

We thank Reviewer#1 for the positive comments regarding the revised manuscript.

*Comment*

Figure 4: ‘over’ should be ‘cover’?

*Response*

Thank you for pointing out this inadvertent error. We have revised ‘over’ to ‘cover’ (Figure 4).

*Comment*

Line 264: it should be ‘the construction of a system’

*Response*

Thank you for this helpful suggestion. We have modified the sentence ‘the construction a system’ to ‘the construction of a system’ (Line 264).
